# Hyperbaric oxygen treatment for inflammatory bowel disease: a systematic review and analysis

**DOI:** 10.1186/2045-9912-2-6

**Published:** 2012-03-15

**Authors:** Daniel A Rossignol

**Affiliations:** 1 Rossignol Medical Center, 3800 West Eau Gallie Blvd., Melbourne, FL 32934, USA

**Keywords:** Hyperbaric oxygen treatment, Inflammation, Oxidative stress, Inflammatory bowel disease, Crohn's disease, Ulcerative colitis, Biomarkers

## Abstract

**Background:**

Traditionally, hyperbaric oxygen treatment (HBOT) has been used to treat a limited repertoire of disease, including decompression sickness and healing of problem wounds. However, some investigators have used HBOT to treat inflammatory bowel disease (IBD), including Crohn's disease and ulcerative colitis.

**Methods:**

Comprehensive searches were conducted in 8 scientific databases through 2011 to identify publications using HBOT in IBD. Human studies and animal models were collated separately.

**Results:**

Thirteen studies of HBOT in Crohn's disease and 6 studies in ulcerative colitis were identified. In all studies, participants had severe disease refractory to standard medical treatments, including corticosteroids, immunomodulators and anti-inflammatory medications. In patients with Crohn's disease, 31/40 (78%) had clinical improvements with HBOT, while all 39 patients with ulcerative colitis improved. One study in Crohn's disease reported a significant decrease in proinflammatory cytokines (IL-1, IL-6 and TNF-alpha) and one study in ulcerative colitis reported a decrease in IL-6 with HBOT. Adverse events were minimal. Twelve publications reported using HBOT in animal models of experimentally-induced IBD, including several studies reporting decreased markers of inflammation or immune dysregulation, including TNF-alpha (3 studies), IL-1beta (2 studies), neopterin (1 study) and myeloperoxidase activity (5 studies). HBOT also decreased oxidative stress markers including malondialdehyde (3 studies) and plasma carbonyl content (2 studies), except for one study that reported increased plasma carbonyl content. Several studies reported HBOT lowered nitric oxide (3 studies) and nitric oxide synthase (3 studies) and one study reported a decrease in prostaglandin E_2 _levels. Four animal studies reported decreased edema or colonic tissue weight with HBOT, and 8 studies reported microscopic improvements on histopathological examination. Although most publications reported improvements with HBOT, some studies suffered from limitations, including possible publication and referral biases, the lack of a control group, the retrospective nature and a small number of participants.

**Conclusions:**

HBOT lowered markers of inflammation and oxidative stress and ameliorated IBD in both human and animal studies. Most treated patients were refractory to standard medical treatments. Additional studies are warranted to investigate the effects of HBOT on biomarkers of oxidative stress and inflammation as well as clinical outcomes in individuals with IBD.

## Background

Inflammatory bowel disease (IBD) is a chronic inflammatory disease of the gastrointestinal (GI) tract characterized by chronic and recurrent ulcerations [1], and includes Crohn's disease and ulcerative colitis. IBD is usually accompanied by severe GI symptoms such as diarrhea, bleeding, abdominal pain, weight loss, and anemia. The symptoms of IBD can be intermittent, with periods of exacerbations and periods that may be relatively free of symptoms. Recent evidence suggests that the pathophysiology of IBD involves immune dysregulation, genetic susceptibilities, intestinal barrier dysfunction, and alterations in microbial flora [2]. Activated macrophages appear to play a key role in the disease process and produce proinflammatory cytokines, including TNF-α and interleukins (IL-6 and IL-8) [3]. Intestinal nitric oxide (NO) levels are also increased in some patients with IBD which may lead to increased intestinal tissue injury [4]. Oxidative stress and mitochondrial dysfunction are also found in some patients with IBD [5,6]. Some investigators have reported that certain infections such as Mycobacterium avium subspecies paratuberculosis may also play a role in IBD [7]. Interestingly, decreased blood flow to the rectum has been reported in some individuals with ulcerative colitis [8].

Current medical treatments for IBD are aimed at maintaining clinical remission and include biologic therapies (e.g., monoclonal antibodies), immunomodulators, aminosalicylates, corticosteroids and other anti-inflammatory modalities [9]. Several studies have reported improvements using hyperbaric oxygen treatment (HBOT) in some patients with IBD [10-12]. HBOT involves inhaling 100% oxygen at greater than one atmosphere absolute (ATA) in a pressurized chamber [13]. HBOT has been used successfully in humans at varying pressures to treat a range of conditions. Many clinical applications of HBOT are at higher pressures (e.g., 2.0 ATA and above) including treatment of decompression sickness, arterial gas embolism, and carbon monoxide poisoning [14]. HBOT has been shown to increase the oxygen content of plasma [15] and body tissues [16] and may normalize oxygen levels in ischemic tissues [17]. Recently, evidence has accumulated that HBOT also has potent anti-inflammatory effects [18-20]. This manuscript is a systematic review and analysis of the medical literature concerning the use of HBOT in IBD.

## Methods

### Search strategy

**Figure 1:**
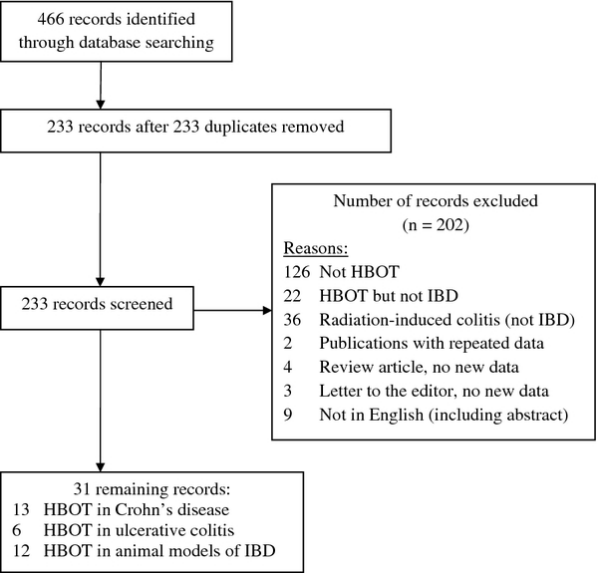
**Flow diagram of studies**.

A search of the Pubmed, EMBASE, Google Scholar, CINAHL, ERIC, AMED, PsychInfo, and Web of Science databases from their inception through December 31, 2011 was conducted to identify and collate pertinent publications using the search terms "hyperbaric oxygen", "HBOT", "hyperbaric" in all combinations with "IBD", "inflammatory bowel", "inflammatory bowel disease", "colitis", "ulcerative colitis", "Crohn", "Crohn's", "esophagitis", "gastritis", "duodenitis", "jejunitis", "ileitis", and "proctitis." Figure 1 demonstrates the flow chart of publications identified by the literature search.

### Study selection

Publications were initially included if they: (1) involved individuals or specimens from individuals with IBD (including Crohn's disease and ulcerative colitis) or were animal models of IBD, and (2) reported using HBOT. Abstracts of identified publications were reviewed to determine if a publication should be included. If the abstract was obscure or missing, the publication was reviewed to determine if inclusion was warranted. Publications of animal models were collated separately. Studies of gastrointestinal abnormalities caused exclusively by radiation treatment were excluded (36 studies). Studies not written in English (9 studies) [21-29] were excluded (unless an English abstract was available). Studies that were purely review articles (4 studies) [30-33] or letters to the editor (3 publications) [34-36] that did not present any new or unique data were also excluded. Finally, studies that published repeated data and not new or unique data (2 studies) [37,38] were excluded.

## Results

### Publications identified by the search

A total of 466 publications were identified. After 233 duplicates were removed, 233 publications were examined. Studies meeting inclusion criteria included 13 publications on the use of HBOT in Crohn's disease, 6 on ulcerative colitis and 12 on animal models of IBD.

### Studies on Crohn's disease

Table 1 outlines the 13 studies [10,11,39-49] meeting inclusion criteria that reported the use of HBOT in Crohn's disease. Six studies were prospective [10,41-43,47,49] and one contained a control group [49]. In each of the 13 studies, the patients had severe Crohn's disease that was refractory to standard medical treatments, including, in some cases, corticosteroids, sulfasalazine, metronidazole, 6-mercaptopurine, and an elemental diet. One study reported improvements with a stay of up to 3 weeks at the Dead Sea (equivalent to 1.05 ATA) in 6 patients [42]. Two studies contained patients from previous studies but reported new data on these patients [40,49]. One study reported the effects of HBOT on flow-mediated vasodilation of the brachial artery in 2 patients with Crohn's disease, but did not report the clinical outcomes of HBOT on gastrointestinal inflammation in these patients [47]. The remaining 9 studies [10,11,39,41,43-46,48] totaled 40 unique patients with Crohn's disease who were treated with HBOT ranging from 2.0 to 2.8 ATA. Of these 40 patients, 31 (78%) had clinical improvements in Crohn's disease with HBOT. One study reported a significant decrease in proinflammatory cytokines (IL-1, IL-6 and TNF-α) after HBOT in 7 patients with Crohn's disease [49].

**Table 1:** Studies of HBOT in Crohn's disease

Author, year, country	Type of study	Number of patients improved/number treated	Location of Crohn's	HBOT parameters	Side effects	Comments/outcomes
Brady et al. 1989 [39], USA	Case report	1/1	Perineal, cutaneous	2.4 ATA 100% oxygen; 6 days a week; 2 h sessions; 67 total sessions	Blurred vision, resolved	Crohn's disease was refractory to surgery and medical treatment (corticosteroids, sulfasalazine, metronidazole, and 6-mercaptopurine) for 8 years; complete and dramatic healing in 2.5 months with HBOT; patient needed additional HBOT over 11 months, then had lasting improvements

Brady 1993 [40], USA	Letter to editor/case report	1/1†	Perineal, cutaneous	Not reported, but presumably the same as previous report (Brady et al. 1989) [39]	NR	Update on patient from previous case report (Brady et al. 1989) [39]. Patient had two additional courses of HBOT (29 and 26 sessions) and was in remission for over 3 years at time of letter

Colombel et al. 1995 [41], France	Prospective, uncontrolled	6/10	Perineal	2.5 ATA 100% oxygen; 2 sessions per day; 5 sessions per week; 40 planned sessions over 4 weeks; 8 patients completed at least 30 treatments	1 patient had bilateral ear drum perforation; another had psychological intolerance	All patients had severe Crohn's disease and had failed one or more standard medical treatments; 2 patients stopped treatments after a few sessions due to side effects; 6 of 8 fully treated patients had partial or complete healing

Fraser and Niv 1995 [42], Israel	Prospective, uncontrolled	6/6	Perianal; ileocolonic	Equivalent to 1.05 ATA	NR	6 patients with Crohn's disease unresponsive to standard medical treatments spent up to 3 weeks at the Dead Sea; significant healing noted in all 6 patients

Iezzi et al. 2011 [43], Brazil	Prospective, uncontrolled	11/14	Perineal or cutaneous	2.4 ATA; 2 h sessions; 1 session per day; 10-50 total sessions	NR	Patients had Crohn's disease refractory to standard medical treatments; 11 of 14 (79%) had "satisfactory improvement" (complete or partial improvement) with HBOT

Jiang et al. 2000 [44], USA	Case report	1/1	ileocolonic	2.5 ATA 100% oxygen; 90 min sessions; 28 day duration	NR	Patient had Crohn's disease and Fournier gangrene; good outcome with surgery, medication and HBOT

Kiel et al. 2011 [45], Australia	Case report	1/1	Cecal	NR	NR	Patient had Crohn's disease and Clostridium septicum infection, treated with antibiotics and HBOT postoperatively with improvements noted

Lavy et al. 1994 [10], Israel	Prospective, uncontrolled	8/10	Perianal	2.5 ATA 100% oxygen; 90 min sessions; 6 times per week; 20 total treatments; HBOT could be repeated for total of 40 sessions	none	10 patients with Crohn's disease refractory to standard medical treatments; improvement observed in 8 of 10 patients; 6 patients had complete healing

Nelson et al. 1990 [46], USA	Case report	1/1	Perineal	2.0-2.8 ATA; 90-120 min sessions; total of 62 sessions	NR	Patient had severe refractory Crohn's disease (failed sulfasalazine and corticosteroids), complete healing with HBOT; no reoccurrence in 24 months after HBOT

Saglam et al. 2008 [47], Turkey	Prospective, uncontrolled	14, 2 had Crohn's disease	NR	2.5 ATA; 90 min sessions; 1 treatment per day	NR	Study measured flow-mediated vasodilation of brachial artery; clinical outcomes of HBOT on GI abnormalities in 2 patients with Crohn's disease not reported

Sipahi et al. 1996 [48], Brazil	Case report	1/1	Perianal	2.4 ATA; 90 min sessions; 7 times per week (1^st ^2 weeks) then 3 times per week; 45 total sessions over 3 months	NR	Complete healing of perianal Crohn's disease with HBOT and antibiotics

Takeshima et al. 1999 [11], Japan	Case report, letter to editor	1/1	Colonic, rectal	2.8 ATA 100% oxygen; 120 min sessions; 20 total sessions	NR	Patient had refractory Crohn's disease (failed prednisolone, sulfasalazine and elemental diet); complete healing of rectal ulcer (by endoscopic examination) with HBOT; clinical remission for 7 months at time of publication

Weisz et al. 1997 [49], Israel	Prospective, controlled (10 healthy controls)	5/7† [same patients as (Lavy et al. 1994)] [10]	Perianal	2.5 ATA 100% oxygen; 90 min sessions; 20-40 total sessions	NR	Complete healing in 3 patients after 20 sessions and in 2 patients after 40 sessions; partial improvements observed in remaining 2 patients. Proinflammatory cytokines significantly decreased during HBOT (IL-1, p < 0.01; IL-6, p < 0.05; TNF-α, p < 0.05)

*NR *not reported

† contained patient(s) from a previous study

### Studies on ulcerative colitis

Table 2 outlines the 6 studies [12,50-54] meeting inclusion criteria that reported the use of HBOT in ulcerative colitis. In each study, the patients had severe ulcerative colitis that was refractory to standard medical treatments, including, in some cases, corticosteroids, 6-mercaptopurine, mesalamine, tetracycline, 5-amino salicylic acid, and azathioprine. One case series [50] reported two patients with ulcerative colitis who improved with HBOT; one of these patients had previously been reported [52]. One study from Bulgaria was not published in English, except for the abstract which reported that 34 patients with chronic ulcerohemorrhagic colitis had improvements with HBOT [53]; however, since only the abstract was written in English, the strengths and weaknesses of this study could not be adequately assessed. The remaining 5 studies [12,50-52,54] totaled 5 unique patients with all 5 reported as having improvements in ulcerative colitis with HBOT; each patient was treated at 2.0 ATA. One study reported a decrease in the IL-6 concentration with HBOT [54].

**Table 2:** Studies of HBOT in ulcerative colitis

Author, year, country	Type of study	Number of patients improved/number treated	HBOT parameters	Side effects	Comments/outcomes
Buchman et al. 2001 [12], USA	Case report	1/1	2.0 ATA 100% oxygen; 2 h duration; 5 days per week; 30 total treatments	NR	Pancolonic ulcerative colitis refractory to conventional medical treatments (corticosteroids for 22 months, 6-mercaptopurine, mesalamine and tetracycline); significant improvements and clinical remission with HBOT (effect lasted 2 months)

Demirturk et al. 2002 [50], Turkey	Case series†	2/2; one from (Hulagu et al. 1997) [52]	2.0 ATA 100% oxygen; 2 h duration; 30 days of treatment	none	Pancolitis in 2 patients with ulcerative colitis was refractory to standard medical treatments (mesalamine and prednisolone); in the first patient during the third week of HBOT, significant improvements were observed; in second patient, improvements observed within 2 weeks; resolution of bloody diarrhea noted in both cases

Gurbuz et al. 2003 [51], Turkey	Case report; letter to editor	1/1	2.0 ATA 100% oxygen; 2 h duration; 35 days of treatment	NR	Left-sided ulcerative colitis was refractory to standard medical treatments (5-amino salicylic acid, methylprednisolone and azathioprine); improvements and clinical remission observed with HBOT after 2 weeks of treatment; remission lasted for at least 6 months

Hulagu et al. 1997 [52], Turkey	Case report	1/1	2.0 ATA 100% oxygen; 2 h duration; 1 treatment per day for 30 days	none	Patient had ulcerative pancolitis; exacerbation was refractory to standard medical treatments (mesalazine and total parenteral nutrition); by third week of HBOT, "definite improvement" observed

Karkumov et al. 1991 [53], Bulgaria	Case series††	34/34	10-12 treatments at 60-75 min; other parameters not reported	NR	All patients had chronic ulcerohemorrhagic colitis; all patients improved after first 5-6 treatments

Kuroki et al. 1998 [54], Japan	Case report	1/1	2 ATA; 60 min duration; 1 treatment per day; 27 days	none	Patient had refractory ulcerative colitis and toxic megacolon (failed antibiotics and intravenous prednisolone); IL-6 dropped from 13.2 to 7.2 pg/ml after 1 HBOT session; significant clinical improvements after third day of HBOT

*NR *not reported

† contained patient(s) from a previous study

†† only the abstract was in English

### Adverse events in Crohn's disease and ulcerative colitis

Of the 13 studies in Crohn's disease, 10 did not report if there were any adverse events [11,40,42-49]. One study reported that one patient had a bilateral ear drum perforation and another had psychological intolerance; both patients had to stop HBOT after a few sessions [41]. It should be noted that ear drum perforation and psychological intolerance are related to changes in pressure and confinement and not side effects of the oxygen being administered. Another study reported blurred vision in one patient which resolved spontaneously [39]. The final study in Crohn's disease specifically reported that there were no adverse events [10]. Of the 6 studies in ulcerative colitis, 3 studies did not report if there were any adverse events [12,51,53] while 3 studies specifically reported that there were no adverse events [50,52,54].

### Studies of animal models of IBD

Table 3 outlines the 12 studies [55-66] meeting inclusion criteria that reported the use of HBOT in experimentally-induced colitis in an animal model of IBD. All 12 studies used rats as the animal model. One study was published only in abstract form [62] and another study was not published in English except for the abstract [65]. Most studies were published in Turkey except for two studies published in the United States [63,66] and one in Israel [64]. Several studies reported that HBOT reduced markers of inflammation, including TNF-alpha [61,63,66], IL-1β [63,66] and neopterin levels [56]. HBOT also decreased edema or colonic tissue weight in 4 studies [56,57,62,64]. One study reported HBOT was equivalent in anti-inflammatory effect to dexamethasone [57]. Myeloperoxidase activity (an index of the accumulation of neutrophils) was reduced after HBOT in 5 studies [55,57,63,64,66]. Some studies reported that HBOT lowered markers of oxidative stress, including malondialdehyde [56,60,61] and plasma carbonyl content [55,61], except for one study that reported HBOT increased protein carbonyl content [65]. Two studies reported HBOT significantly increased glutathione peroxidase and superoxide dismutase levels [60,61]. HBOT also decreased nitric oxide [58,61,63] and nitric oxide synthase levels [63,64,66]. Finally, in one study, HBOT decreased prostaglandin E_2 _levels [64]. Nine studies used histopathology scores to document changes with HBOT [55-61,64,65] and 8 reported microscopic improvements [56-61,64,65].

**Table 3:** Studies of HBOT in animal models of experimentally-induced colitis

Author, year, country	Animal used	Number of animals	HBOT parameters	Comments/outcomes
Akin et al. 2002 [55], Turkey	Rat	42	2.0 ATA 100% oxygen; 2 h treatments for 2 (acute) or 14 (chronic) days	HBOT studied in acute (2 days) and chronic (14 days) colitis; compared to sham treatment, HBOT significantly ameliorated macroscopic, but not microscopic, damage in chronic colitis but not acute colitis; HBOT also significantly reduced myeloperoxidase activity (an index of the accumulation of neutrophils) in acute colitis and decreased plasma carbonyl content (a marker of oxidative damage) in chronic colitis

Altinel et al. 2011 [56], Turkey	Rat	40	2.8 ATA 100% oxygen; 90 min treatments; 2 treatments per day for 5 days	Compared to sham treatment, HBOT significantly reduced the severity of colitis as measured by a histopathological score; HBOT also significantly reduced malondialdehyde (a marker of oxidative stress) and neopterin (a marker of cell-mediated immune activation).

Atug et al. 2008 [57], Turkey	Rat	48	2.0 ATA 100% oxygen; 75 min in duration; 2 treatments per day for up to 3 days	Compared to sham treatment, HBOT significantly decreased colitis on microscopic, macroscopic and tissue weight testing; HBOT also significantly decreased myeloperoxidase activity; HBOT was equivalent to dexamethasone in anti-inflammatory effect

Ercin et al. 2009 [58], Turkey	Rat	36	2.4 ATA 100% oxygen; 1 h duration; 2 treatments per day for 7 days	Compared to sham treatment, HBOT significantly decreased colitis on both microscopic and macroscopic testing compared to control group and prevented weight loss; HBOT also significantly reduced nitric oxide levels

Gorgulu et al. 2006 [59], Turkey	Rat	50	2.8 ATA 100% oxygen; 90 min duration; 2 treatments per day for 3 days	Compared to sham treatment, HBOT significantly reduced histopathologic score of inflammation; HBOT slightly reduced myeloperoxidase activity but not significantly

Gulec et al. 2004 [60], Turkey	Rat	36	2.5 ATA; 90 min duration; 2 treatments per day for 5 days	HBOT significantly reduced malondialdehyde levels in erythrocytes, plasma and intestinal tissue; HBOT significantly increased glutathione peroxidase and superoxide dismutase levels; HBOT significantly improved histopathological scores

Guven et al. 2009 [61], Turkey	Rat	30	2.8 ATA 100% oxygen; 90 min duration for 3 days	HBOT significantly reduced malondialdehyde levels, nitric oxide levels, TNF-alpha levels, and protein carbonyl content; HBOT significantly increased glutathione peroxidase and superoxide dismutase levels; HBOT significantly reduced histological evidence of intestinal injury

Guven et al. 2010 [62], Turkey†	Rat	40	2.8 ATA 100% oxygen; 90 min duration; 2 treatments per day for 4 days	HBOT reduced malondialdehyde levels (non-significantly); HBOT decreased inflammation and edema compared to controls

Nandi et al. 2010 [63], USA	Rat	NR	2.3 ATA 100% oxygen; 1 h duration for 2-5 days	HBOT significantly decreased indomethacin-induced ulceration and reduced TNF-α, IL-1β, nitric oxide, nitric oxide synthase levels as well as myeloperoxidase activity

Rachmilewitz et al. 1998 [64], Israel	Rat	56	2.4 ATA 100% oxygen; 1 or 7 days in duration	HBOT significantly decreased colonic tissue weight, myeloperoxidase levels, Prostaglandin E_2 _generation and nitric oxide synthase activity; HBOT significantly decreased colitis on histological examination

Simsek et al. 2011 [65], Turkey††	Rat	20	2.5 ATA 100% oxygen; 60 min duration	HBOT significantly decreased intestinal injury as measured by an apoptosis score and significantly increased protein carbonyl content

Yang et al. 2006 [66], USA	Rat	48	2.3 ATA 100% oxygen; 60 min duration; 1-2 treatments per day for 2 or 5 days	HBOT significantly decreased TNF-α and IL-1β; HBOT significantly reduced intestinal ulceration; HBOT significantly reduced myeloperoxidase and nitric oxide synthase activities

*NR *not reported

† publication was only in abstract form

†† only the abstract was in English

## Discussion

Crohn's disease and ulcerative colitis are forms of IBD which have a limited repertoire of treatment options. Management consists of maintaining clinical remission. In the reviewed studies, HBOT was associated with improvements in most treated patients. Of note, all of the patients in these studies had IBD that was refractory to standard medical treatments and HBOT was essentially used as a treatment of last resort. Adverse events were minimal in most studies. Several animal studies reported that HBOT decreased inflammation and oxidative stress markers and led to improvements in IBD on both a microscopic and macroscopic level.

### Effects of HBOT on inflammation and immune dysregulation in IBD

IBD is characterized by inflammation, ulcerations and the accumulation of neutrophils. Many of the reviewed animal models replicated IBD by inducing colitis which was accompanied by intestinal edema, ulcerations, accumulation of neutrophils, increased nitric oxide levels and elevated cytokines. Therefore, these animal models appeared effective at reproducing the pathophysiology of IBD.

HBOT has been shown to possess potent anti-inflammatory properties in both animal [55,67,68] and human studies [10,11,20,46,69] and has been reported to decrease the production of pro-inflammatory cytokines (such as TNF-alpha, interferon-gamma, IL-1 and IL-6) in both animal [66,70] and human studies [20,49] as well as increase IL-10 levels [71]. Several of the reviewed studies (including human studies and animal models) reported a decrease in inflammation as measured by reduced tissue edema and histopathological changes, as well as a decrease in TNF-alpha, IL-1 and IL-6 with HBOT [49,54,61,63,66]. Some of these effects may have been mediated, to some degree, by the activity of stem cells [72]. Stem cells have been shown to migrate to the sites of inflammation and damage [73]. Several studies have reported that HBOT can stimulate the growth and differentiation of stem cells [74-78] as well as mobilize stem cells into the circulation from bone marrow [79,80]. A number of studies have reported improvements using stem cells in some patients with Crohn's disease [81-86] or ulcerative colitis [84]. Interestingly, some animal studies report that the use of HBOT combined with stem cells was more effective than stem cells alone [87-90]. Therefore, HBOT may help lower inflammation in patients with IBD through the increased mobilization of stem cells.

Animal models of IBD demonstrate that the GI mucosa develops hypoxia [91]. Hypoxia has been reported to increase oxidative stress and inhibit mitochondrial function [92] as well as increase inflammation [93]. Chronic inflammation can lead to tissue edema and impaired oxygen extraction from the blood into tissue [94] and a vicious cycle between hypoxia and inflammation can therefore ensue [95]. Hypoxia also causes an increase in hypoxia-inducible transcription factor (HIF) which, in turn, can initiate an inflammatory cascade [96,97]. In fact, HIF is essential for inflammation mediated by myeloid cells [98] and rats null for HIF demonstrate almost complete inhibition of the inflammatory response [99]. Furthermore, intestinal biopsies from patients with IBD show elevated levels of HIF in the mucosa [100]. Hypoxia and increased levels of HIF can then activate nuclear factor *κ*B (NF-*κ*B) which subsequently stimulates the production of TNF-alpha [101]. HBOT has been shown to inhibit the expression of HIF and its target genes [102]. Therefore, another method whereby HBOT may lower inflammation in patients with IBD is by the relief of hypoxia and the inhibition of HIF expression [95]. Interestingly, decreased blood flow to the rectum has been reported in some individuals with ulcerative colitis [8]. A reduction in tissue edema with the use of HBOT might lead to improved blood supply and relief of hypoxia. Therefore, HBOT may also ameliorate hypoxia and inflammation in individuals with IBD by reducing tissue edema.

Finally, the inflammation found in IBD could be secondary to an infectious agent. Since some investigators have reported that Mycobacterium avium subspecies paratuberculosis may play a role in IBD [7], it is possible the HBOT may be beneficial as it has been reported to kill Mycobacterium species [103,104]. Additional studies investigating the effects of HBOT on inflammation and biomarkers of inflammation in individuals with IBD are warranted.

### Effects of HBOT on oxidative stress in IBD

Some studies have reported evidence of oxidative stress and mitochondrial dysfunction in individuals with IBD. Several of the animal models of IBD reported a reduction in oxidative stress markers with HBOT. It should be noted that, theoretically, HBOT might increase oxidative stress through the augmented production of reactive oxygen species (ROS) from the high concentration of oxygen [105]. This may occur because increased oxygen delivery to mitochondria can increase ROS production. However, HBOT has also been shown to upregulate the production of antioxidant enzymes such as superoxide dismutase [106,107], glutathione peroxidase [60], catalase [108], paraoxonase [109] and heme-oxygenase 1 [110,111]. This increase in antioxidant enzyme levels has been termed "conditioning" and can protect against damage caused by ROS [95,112]. Two animal models of IBD reported a significant increase in glutathione peroxidase and superoxide dismutase levels with HBOT [60,61], suggesting that the increased production of antioxidant enzymes may have limited or lowered oxidative stress in these studies. Although none of the reviewed studies measured changes in mitochondrial function with HBOT, it is possible that HBOT may have led to some improvements by augmenting mitochondrial function as reported in previous studies [113-116]. Further studies examining the effects of HBOT on oxidative stress and mitochondrial dysfunction in individuals with IBD are needed.

### Effects of HBOT on IBD in human studies

The total number of identified publications utilizing HBOT in Crohn's disease was about twice the number of studies in ulcerative colitis. In these studies, the number of patients treated in both diseases was similar, although one study in ulcerative colitis reported improvements in 34 patients, but this finding was only reported in an English abstract, and therefore the strengths and weaknesses of this study could not be adequately assessed [53]. The remaining 5 studies in ulcerative colitis only totaled 5 patients. Therefore, the evidence in the reviewed studies for a positive effect of HBOT is stronger for Crohn's disease than for ulcerative colitis.

In the studies of Crohn's disease, 78% of treated patients had an improvement with HBOT at a pressure ranging from 2.0 to 2.8 ATA. In the studies of ulcerative colitis, all treated patients showed improvements with a pressure delivered at 2.0 ATA. These studies suggest that a higher pressure may be needed to achieve these improvements. However, because none of these studies utilized a lower pressure of HBOT, it is not known if a lower pressure or oxygen level would be beneficial in IBD. However, some investigators have reported improvements in GI function in some children with autism using HBOT at 1.3 to 1.5 ATA [117,118]. Furthermore, previous studies have reported improvements in certain neurological conditions using hyperbaric treatment at lower pressures and/or oxygen levels [119-122]. Additional studies using HBOT at varying oxygen concentrations and atmospheric pressures would be helpful in determining optimal treatment protocols.

It is especially noteworthy that all of the studies on Crohn's disease and ulcerative colitis used HBOT in patients who were refractory to multiple medical treatments, including, in some cases, corticosteroids, 6-mercaptopurine, mesalamine, tetracycline, 5-amino salicylic acid, azathioprine, sulfasalazine, metronidazole, and an elemental diet. It is possible that earlier treatment with HBOT in individuals with IBD before severe symptoms develop would be even more effective in ameliorating the condition. Additional studies examining the clinical effects of HBOT in individuals with IBD would be helpful in assessing this possibility.

### Limitations

Many of the reviewed studies suffered from limitations, including the lack of a control group, the open-label nature and the small number of participants. Some of the studies were retrospective while only a few were prospective [10,41-43,47,49]. Most of the reviewed studies did not report if HBOT was supplied in a monoplace or multiplace chamber, and whether or not masks or hood systems were used, and therefore the ability to determine the effects of different types of chambers or oxygen delivery systems in these studies could not be adequately assessed. Most studies did not specifically report if there were any adverse events. Presumably, if there had been any adverse events, the investigators would have reported them. Therefore, the adverse events in these studies were most likely minimal, but since most studies did not report this, a full assessment cannot be performed. Some of the studies may have been limited by referral bias; for example, none of the studies were population-based which could have reduced potential referral bias. Another limitation of the studies may have been publication bias where only cases that had improvements with HBOT were published, and cases of IBD that were treated with HBOT that did not improve may not have been published. However, it should be noted that for a new treatment to be recognized, it is common for case reports to first be published to introduce and confirm a new treatment before sufficient interest is generated to commit resources to completing larger high-quality, stronger studies. Since most reviewed studies had limitations, larger studies examining the effects of HBOT in individuals with IBD are warranted.

## Conclusions

HBOT ameliorated symptoms of IBD in both human studies and animal models. HBOT also lowered pro-inflammatory cytokine concentrations and lowered biomarkers of inflammation and oxidative stress. Adverse events were minimal. Many studies suffered from limitations, including possible publication and referral biases, the lack of a control group, the retrospective nature and a small number of participants. Additional studies are warranted to investigate both the effects of HBOT on biomarkers of oxidative stress and inflammation as well as clinical outcomes in individuals with IBD.

## Abbreviations

ATA: Atmosphere absolute; GI: Gastrointestinal; HBOT: Hyperbaric oxygen treatment; IBD: Inflammatory bowel disease; NO: Nitric oxide.

## Competing interests

The author treats individuals with HBOT in his clinical practice and derives revenue from this. He has previously received research funding from the International Hyperbarics Association (IHA) for two studies of hyperbaric treatment in children with autism [120,121] and is a medical advisor (unpaid) for IHA.
